# The Effect of Ocular Perfusion Pressure on Retinal Thickness in Young People with Presumed Systemic Hypotension

**DOI:** 10.3390/vision5030036

**Published:** 2021-07-14

**Authors:** Naazia Vawda, Alvin Munsamy

**Affiliations:** Alvin Jeffrey Munsamy, Room E5-642, Discipline of Optometry, 6th Floor, E Block, Westville Campus, University Road, Westville, Durban 3629, South Africa; munsamya1@ukzn.ac.za

**Keywords:** systemic hypotension, glaucoma, ocular perfusion pressure, ganglion cell complex, retinal nerve fiber layer

## Abstract

Low ocular perfusion pressure (OPP) may increase the risk of optic neuropathy. This study investigated the effects of OPP on the ganglion cell complex (GCC) and optic nerve head-retinal nerve fibre layer (ONH-RNFL) thickness in presumed systemic hypotensives (PSH). Fifteen participants with PSH and 14 controls underwent automated sphygmomanometry and Icare tonometry to calculate OPP: mean OPP (MOPP), systolic OPP (SOPP), and diastolic OPP (DOPP). ONH-RNFL and macula GCC thickness were evaluated using the Optovue iVue optical coherence tomographer. Statistical analysis comprised independent *t*-tests, the Mann–Whitney U test and binary logistic regression analysis. There was no significant difference when comparing ONH-RNFL and macula GCC thickness between both groups. Increased MOPP (OR = 0.51; 95% CI: 0.27–0.97; *p* = 0.039) and SOPP (OR = 0.79; 95% CI: 0.64–0.98; *p* = 0.035) were significantly associated with a decreased risk of reductions in GCC total thickness. Increased SOPP (OR = 0.11; 95% CI: 0.01–0.89; *p* = 0.027) was significantly associated with a decreased risk of reductions in the average ONH-RNFL thickness. The study found no significant retinal thickness changes in PSH’s, in comparison to the controls. The study established that, by increasing MOPP and SOPP, there was a decreased risk of reductions in the total GCC thickness and average ONH-RNFL thickness. Higher SOPP may decrease the possibility of retinal thinning of the GCC and ONH-RNFL. However, higher MOPP may decrease the odds of thinning of the GCC before ONH-RNFL changes.

## 1. Introduction

Glaucoma is the leading cause of irreversible blindness worldwide [1]. The estimated global prevalence of glaucoma is 3.54%, with the highest prevalence in Africa [1]. To highlight the global burden of glaucoma, by 2010, 1 out of 15 blind people were blind due to glaucoma, and 1 in 45 visually impaired people were visually impaired due to glaucoma [2]. The number of people with glaucoma will increase to 111.8 million by 2040, affecting those in Asia and Africa disproportionately [1]. Glaucoma is defined as a heterogeneous group of chronic neurodegenerative disorders. It is a selective and progressive optic neuropathy that is characterised by the accelerated death of retinal ganglion cells (RGC) and their retinal nerve fibre layer (RNFL) axons leading to visual field loss [3,4,5]. It is chronic, imperceptible and is difficult to diagnose, especially in asymptomatic patients, thus making timely diagnosis and evaluation of the disease through structural and functional analysis significant [6]. The mechanism of glaucoma is not clearly understood, particularly with extraretinal neurodegeneration [7].

The interaction between systemic blood pressure (BP) and intraocular pressure (IOP) can be related as ocular perfusion pressure (OPP) [8]. Low blood pressure and/or high IOP may result in reduced OPP [9]. Reduced OPP may indicate changes in blood flow and induce ischemia to the retina [10]. OPP is categorised into systolic ocular perfusion pressure (SOPP), diastolic ocular perfusion pressure (DOPP), and mean ocular perfusion pressure (MOPP) and is derived by using equations that calculate the difference between systemic blood pressure (BP) and intraocular pressure (IOP). SOPP is calculated as systolic BP minus IOP; DOPP is calculated as diastolic BP minus IOP and MOPP is calculated as (1/3 systolic BP + 2/3 diastolic BP) minus IOP [11,12]. A decrease in OPP could result in the thinning of the RNFL and ganglion cell layer (GCL), which, in turn, predisposes glaucoma suspects to the accelerated thinning of the inner retina.

Low OPP, calculated using the abovementioned equations, has proven to be an important factor in assessing the development and progression of glaucoma as shown in the Los Angeles Latino Eye Study [11], the Barbados Eye study [12] and the Baltimore Eye Survey [13]. The Los Angeles Latino Eye Study [11] found that participants at the extremes of blood pressure and those with MOPP < 50 mmHg, DOPP ≤ 40 mmHg, and SOPP ≤ 80 mmHg, independent of the influence of IOP, were at an increased risk of primary open-angle glaucoma (POAG) [11]. The Barbados Eye Study [12] found the highest prevalence of POAG with MOPP < 42 mmHg, SOPP < 101.3 mmHg and DOPP < 55 mmHg with an increased relative risk for POAG with lower perfusion pressure. The Baltimore Eye Survey [13] found a six times higher risk of developing POAG with decreased perfusion pressure. In systemic hypotension, there is a reduction in blood pressure, which, in turn, reduces OPP. Usually, there are autoregulation mechanisms in effect. However, if BP falls below this critical range, there may be a significant decrease in OPP and blood flow. Glaucoma patients are believed to have abnormalities in the auto-regulatory mechanism [11].

Variations in OPP, combined with a reduced ability to maintain blood flow, increases the risk of a reduced metabolic supply to the retina [8]. Ischemia and/or reperfusion as a result of inadequate or unstable blood supply may result in optic nerve tissue and axonal damage. This vascular mechanism has been associated with glaucomatous optic neuropathy (GON) in people with arterial hypotension, arterial hypertension, vasospasm, and other circulation disorders [10].

The Los Angeles Latino Eye study [11] defined hypotension as systolic blood pressure (SBP) ≤ 90 mmHg and diastolic blood pressure (DBP) ≤ 60 mmHg. The World Health Organisation (WHO) defines systemic hypotension as SBP lower than 100 mmHg in women and 110 mmHg in men regardless of DBP [14]. Since no clinical diagnosis is usually made for systemic hypotension, we have referred to it as presumed systemic hypotension (PSH) in this study. The relationship between low OPP and systemic hypotension in previous studies has led to this study. This study focused on the effects of low OPP on retinal thickness, as a predisposition for optic neuropathy in those with PSH. No previous studies on systemic hypotension and the retina are known to have been done.

Management of glaucoma focuses on lowering IOP, which remains the rational, proven treatment method. However, IOP has a 24-hour circadian rhythm, indicating that IOP undergoes dynamic changes [15]. The most effective procedure for lowering IOP in uncontrolled glaucoma in those with ocular hypertension is surgical intervention. However, it is not easily available to those who need it. Similarly, in conditions that are fully treatable by surgery, such as vision impairment due to uncorrected refractive error and cataracts, more than 75% of patients do not have access [16]. Characteristics of refractory glaucoma include advanced optic nerve damage, severe impairment of the visual field, and uncontrolled intraocular pressure (IOP). These characteristics may be seen despite medical therapy or glaucoma surgery, thus making management of IOP difficult. This indicates that preservation of visual function should be part of our treatment choices [17]. To detect and monitor changes over time in those with systemic hypotension and low OPP, it may be beneficial to assess GCC and RNFL thickness.

Assessing the thickness of the GCC and ONH-RNFL thickness may be valuable in people with systemic hypotension as they may, over time, suffer reduced perfusion, translating into optic neuropathy. Obstructive sleep apnoea syndrome (OSAS) presents with a similar mechanism of reduced perfusion pressure, which affects the retina. Thinning of the RNFL may be caused by the loss of ganglion cells secondary to hypoxia as well as hypoxemia induced by OSAS, which has been thought to be due to profound hypoxia and vascular dysregulation disturbing OPP [18]. This may premise similar concerns that may occur in chronic systemic hypotension. This study aimed to investigate the effect of presumed systemic hypotension on ocular perfusion pressure and its relationship to ONH-RNFL and GCC (macula) thickness. Assessing the innermost retinal layers, viz. the macula GCC and ONH-RNFL, may be a good indicator to monitor hypoxic changes because of reduced OPP. This study is original in its aims as there is a lack of literature on the topic of hypotension and its effects on the retina.

## 2. Methods and Materials

The study is observational in design, using a case-control approach, and was conducted at a university eye clinic in South Africa in 2018 and 2019. The study population comprised 15 presumed systemic hypotensive participants (cases, 30 eyes), who served as the experimental group, and 14 normotensive participants (controls, 28 eyes), who served as the control group, all of whom had no ocular or other systemic comorbidities. Ethical clearance was obtained from the Biomedical Research and Ethics Committee at UKZN.

### 2.1. Sampling

Participants were sourced using a non-probability, purposive sampling technique that was age- and sex-matched for both groups. A projected sample of 15 participants was chosen based on a study involving obstructive sleep apnoea syndrome and prevalence estimates from studies on low systemic hypotension, as no previous studies on systemic hypotension and the retina are known to have been done. The projected sample size was based on the target sample required, assuming the following measures:

Normal (in microns): average superior RNFL thickness of 90.5 ± 10.4 microns, average inferior RNFL thickness of 90.7 ± 10.3 microns. As no other studies investigated systemic hypotension, severe OSAS was substituted for abnormal thickness: average superior RNFL thickness of 73.4 ± 8.8 microns, average inferior RNFL thickness of 76.8 ± 10.6 microns.

The estimated sample size for power (1-β) of 0.8 and at alpha α of 0.5 resulted in 18 normotensives and 18 hypotensive eyes. To detect a smaller difference between normal BP and hypotensive participants, the sample size of 15 (30 eyes) was calculated.

### 2.2. Ethical Considerations

Institutional ethical clearance was obtained before the commencement of the study. Each participant was provided with an information sheet regarding the purpose of the study before informed consent was obtained. The tenets of the declaration of Helsinki were observed for human participation throughout the study.

### 2.3. Selection Criteria

Inclusion:

- Those included in the study were adults with presumed systemic hypotension of SBP <100 mmHg in women and SBP <110 mmHg in men.
- Adults with aided/unaided visual acuity of 6/6.
- Non-glaucomatous participants.

Exclusion:

- Participants with chronic systemic disease and those on any chronic medication.
- Participants with any ocular pathology and those with moderate and high myopia > 5D.
- Smoking, alcohol, caffeine and water consumption were not allowed within 30 min of the screening.

### 2.4. Screening Procedure

The screening of participants involved an ocular and systemic case history, together with a checklist of systemic hypotensive symptoms. Records of any systemic illnesses, such as hypertension and diabetes, as well as ocular conditions such as glaucoma, were noted. Any medication was also noted. A thorough slit lamp examination, ophthalmoscopy, fundus photography, and a visual acuity test were performed to rule out any pathology, including glaucoma. A checklist of the most common symptoms of low BP was recorded. The symptoms which formed the checklist included fatigue, feeling faint, dizziness, loss of concentration, and headaches [19,20,21,22,23,24].

### 2.5. Data Collection

BP and IOP were measured in both groups. In an attempt to factor in diurnal variations, optimal times for BP and IOP readings were obtained during the peak and trough, which is between 9:00 and 10:00 and 15:00 and 16:00 [25,26,27].

#### 2.5.1. Automated Sphygmomanometry (BP)

An average of two BP readings were taken on both arms using the Omron Intelli i7, a validated automated electronic sphygmomanometer. To ensure accurate measurements, certain factors were taken into consideration. Participants were required to rest for three to five minutes at an average room temperature with their back supported and legs uncrossed to ensure the participant was relaxed [26,28]. Participants were required to have emptied their bladder, not consumed alcohol or caffeine, or smoked 30 min before BP measurements were taken [26,28]. The correct cuff size was utilised and placed on the participant’s arm and not over clothing. The arm used for the BP measurement was at heart-level and rested on a table to ensure accurate readings [28]. BP was defined as SBP < 100 mmHg in women and SBP < 110 mmHg in men, regardless of DBP, to satisfy the objective presumption of systemic hypotension. In a population survey by Myers et al. [29], results taken with a manual sphygmomanometer were compared to those taken with an automated sphygmomanometer. Results showed that conventional manual BP readings can be replaced by readings taken by a validated automated sphygmomanometer.

#### 2.5.2. Tonometry (IOP)

IOP was measured using the Icare tonometer during the morning and afternoon sessions as specified above. The normal range of IOP is 10–20 mmHg [30]. The Icare tonometer takes six consecutive readings before a measurement is provided at a significance of *p* < 0.05. “P_” indicates that the standard deviation is higher than normal but the effect is unlikely to be relevant. “P-” also indicates a deviation that is unlikely to be significant but requires a new reading if IOP is greater than 19 mmHg [31]. IOP was measured twice on each eye with an acceptable reading of P_ or P-, and the average was used. The participants were required to have not consumed water before or during testing, as water consumption increases venous pressure and may increase IOP up to 6 mmHg [32]. Body position and stress have also been found to affect IOP readings [32]. Thus, measurements were taken in the seated upright position with the patient having relaxed for three to five minutes before the reading was taken. During the morning session, BP and IOP readings were taken on the right eye first and this was alternated in the afternoon. A new probe was used for each participant to ensure accuracy. A single examiner obtained all the measurements to ensure that a standard procedure was followed. The Goldmann Applanation Tonometer is widely known as the gold standard in assessing IOP [33]. Brusini et al. [33] found no significant difference between IOP readings taken with the IcareTA01I Rebound Tonometer and the Goldmann Applanation Tonometer. Pakrou et al. [34] clinically compared the Icare to the Goldman Applanation Tonometer. A good correlation between the two methods was found even at extremes of IOP.

#### 2.5.3. OPP Calculations (mmHg)

The morning and afternoon readings of both BP and IOP were averaged and used to calculate the OPP using OPP formulae to determine the MOPP, DOPP, and SOPP values as follows [11,12]:

- Systolic ocular perfusion pressure (SOPP) = systolic BP-IOP
- Diastolic ocular perfusion pressure (DOPP) = diastolic BP-IOP
- Mean ocular perfusion pressure (MOPP) = (1/3 SBP + 2/3 DBP)-IOP [11,12]

#### 2.5.4. Retinal Thickness Measurements

All participants were required to undergo further examinations to obtain retinal thickness measurements using the Optovue iVue 100 Spectral Domain OCT (SD-OCT) version 2016.3.0.18. The Optovue iVue100 OCT has an image acquisition rate of 25,000 A-scan/second. The frame rate is 56 to 4096 A-scan/frame with an optical resolution depth of 5 µm and a scan range depth of 2–2.3 mm [35]. The GCC thickness at the macula and the ONH-RNFL thickness in both groups were obtained. An average of three readings for each eye with a scan quality index of 40 or more for both variables was satisfied to ensure reliable scans.

**Figure 1:**
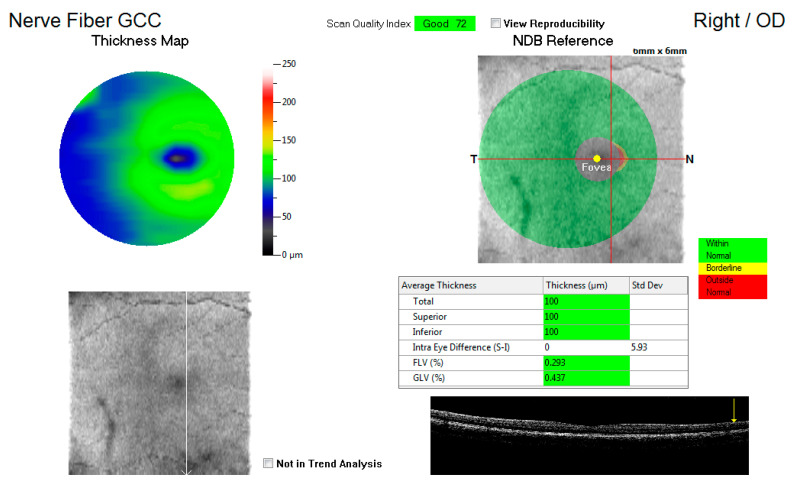
An illustration of the ganglion cell complex (GCC) thickness map at the macula.

Figure 1 shows an example of the GCC map at the macula, displaying the total average thickness as well as the superior and inferior thickness (microns) with a key indicating whether the measurements are within normal, borderline, or outside the normal range. These average thicknesses in microns for the three variables were captured for each eye and compared to the control group.

**Figure 2:**
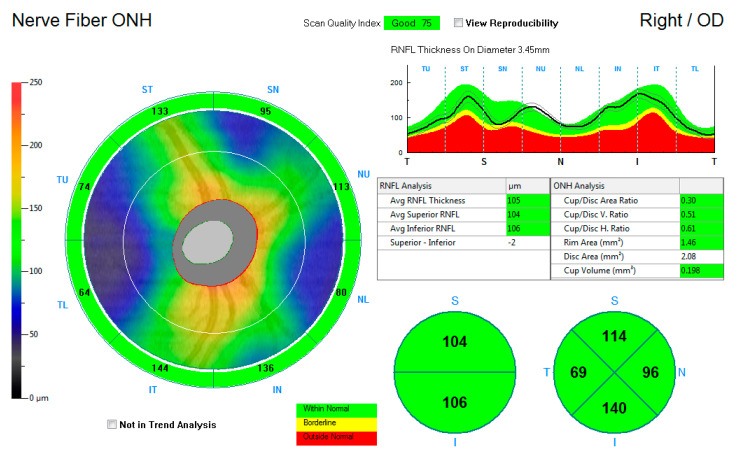
An illustration of the retinal nerve fibre layer thickness map around the optic nerve head.

Figure 2 shows the ONH-RNFL thickness map, reporting the average RNFL thickness and the superior and inferior average hemispheric RNFL thickness. Thickness measurements were further recorded into a quadrantic map for the superior, inferior, nasal, and temporal subfields. The average measurements for the average ONH-RNFL, the inferior hemisphere ONH-RNFL, the superior hemisphere ONH-RNFL, the superior quadrant ONH-RNFL, the inferior quadrant ONH-RNFL, the nasal quadrant ONH-RNFL, and the temporal quadrant ONH-RNFL thicknesses were captured for each eye and compared to the control group.

### 2.6. Data Analysis

Data analysis was performed using STATA 16 and SPSS (Statistical Package for the Social Sciences Version 25.0). The Kolmogorov–Smirnov test was used to test the skewness of the data. Thereafter, the independent *t*-test and/or the Mann–Whitney U-test were used to compare the difference in measurements between the experimental and control groups. A binary logistic regression analysis was used to measure the association between OPP and GCC and RNFL thickness; this relationship is represented by the odds ratios (OR). With OPP being the independent variable and the GCC and RNFL layers being the dependent variable, a relationship between OPP and retinal thickness was established inversely. A *p*-value of less than 0.05 was considered statistically significant for all the tests.

## 3. Results

The experimental group (PSH) comprised 15 females with a mean age of 23.53 ± 6.60 years, with an ethnic composition of six Africans and nine Indians. The control group consisted of 14 females with a mean age of 24.38 ± 7.91 years with an ethnic composition of seven Africans, six Indians, and one Caucasian participant.

Table 1 shows a comparison between the mean IOP, mean systemic BP, and OPP for both groups. An independent *t*-test showed that there was a statistically significant reduction in the experimental group when compared to the control group for both MOPP and SOPP.

Table 2 shows that the comparisons of all mean ONH-RNFL thickness measurements showed no statistically significant difference between the experimental and control groups.

**Figure 3:**
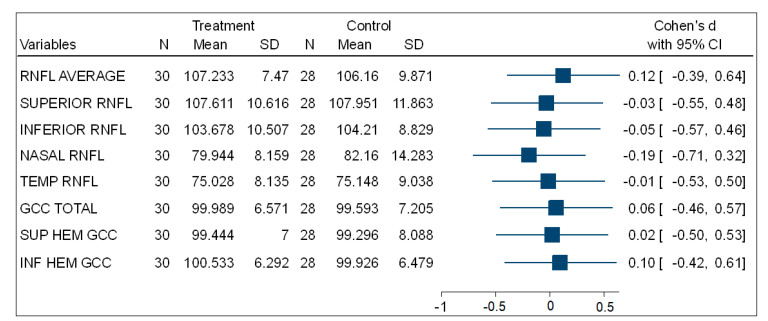
A forest plot showing the effect size of the comparison of (ONH-RNFL) layers and (GCC-macula) layers between the PSH and control groups.

Table 3 shows that, in the comparisons for the mean total, superior, and inferior hemisphere ganglion cell complex thickness (GCC) at the macula, there was no statistically significant difference between the experimental (PSH) and control (normotensive) participants. Figure 3 is a forest plot showing the effect size using Cohen’s d comparing the mean thickness of the ONH-RNFL quadrants and GCC quadrants between the PSH and control groups. This shows very small effect size differences between both groups.

### 3.1. The Association between OPP and GCC Thickness (Macula) for the PSH Group

Table 4 summarises the significant associations, calculated from a multivariate binary logistic regression for retinal thickness and OPP represented by odds ratios (OR). The participants with higher MOPP (OR = 0.51; 95% CI: 0.27–0.97; *p* = 0.039) were negatively associated with the odds of having a decreased total GCC thickness.

**Figure 4:**
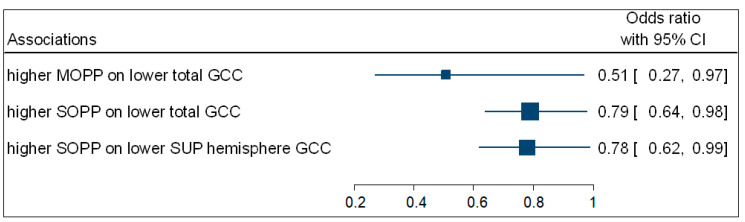
A forest plot showing associations between OPP and macula GCC.

Participants with increased SOPP (OR = 0.79; 95% CI: 0.64−0.98; *p* = 0.035) were negatively associated with the odds of having a lower total GCC thickness. Participants with higher SOPP (OR = 0.78; 95% CI: 0.62−0.99; *p* = 0.049) were also negatively associated with the odds of having a lower GCC superior hemisphere thickness. No associations were observed between DOPP and macula GCC thickness. Figure 4 is a complementary forest plot showing the associations between MOPP and total GCC thickness, as well as the associations between SOPP and total and superior hemisphere GCC thickness.

### 3.2. The Association between OPP and ONH-RNFL Thickness for the PSH Group

**Figure 5:**
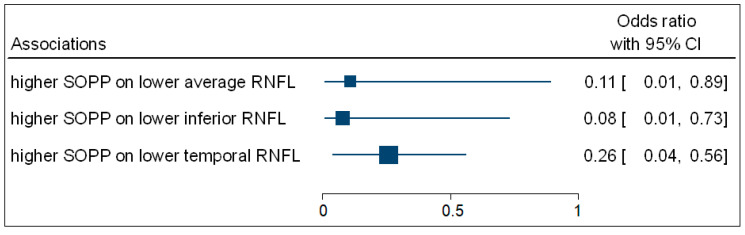
A forest plot showing associations between OPP and ONH-RNFL.

Table 4 shows that the multivariate binary logistic regression indicated that participants with higher SOPP (OR = 0.11; 95% CI: 0.01–0.89; *p* = 0.027) were negatively associated with the odds of having decreased average ONH-RNFL thickness. Participants with higher SOPP (OR = 0.08; 95% CI: 0.01–0.73; *p* = 0.017) were negatively associated with the odds of having decreased inferior ONH-RNFL thickness. Participants with higher SOPP (OR = 0.26; 95% CI: 0.04–0.56; *p* = 0.036) were negatively associated with the odds of having decreased temporal ONH-RNFL thickness. Figure 5 is a complementary forest plot showing the associations between SOPP and average, inferior, and temporal ONH-RNFL thickness. No associations were observed between MOPP and DOPP and ONH-RNFL thickness.

### 3.3. The Association between OPP and Retinal Thickness for the PSH Group

**Figure 6:**
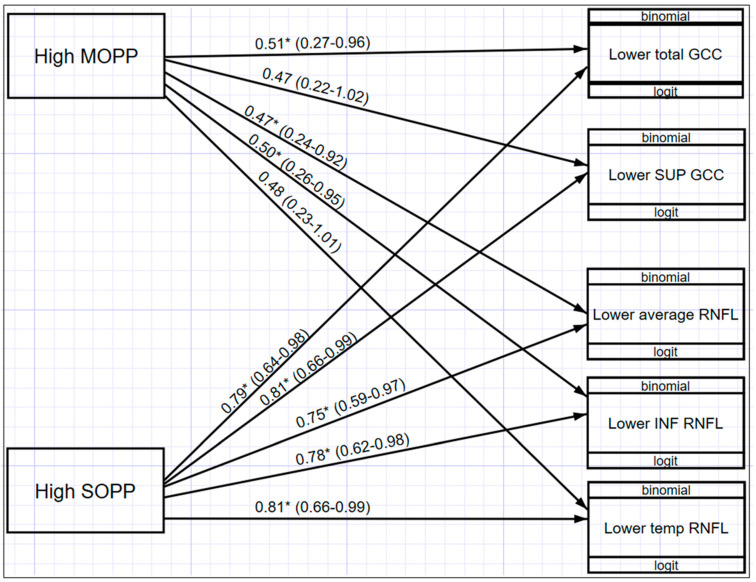
Structural equation model (SEM) with the relationships between OPP and GCC and ONH-RNFL * (*p* < 0.05).

Figure 6 illustrates a multivariate structured equation model (SEM) showing the relationship between OPP and macula GCC and ONH-RNFL for those values that were statistically significant (*p* < 0.05). Participants with higher MOPP are associated with decreased odds of having lower total GCC (OR = 0.51; 95% CI: 0.27–0.96), lower average RNFL (OR = 0.47; 95% CI: 0.24–0.92), and lower inferior RNFL (OR = 0.50; 95% CI: 0.26–0.95) thickness. In addition, MOPP has a significant differential effect across both macula GCC and ONH-RNFL, indicating that the effect of MOPP on total GCC is significantly greater than the effect of MOPP on ONH-RNFL. It was also found that participants with higher SOPP were associated with decreased odds of having lower total GCC (OR = 0.79; 95% CI: 0.64–0.98); lower superior GCC (OR = 0.81; 95% CI: 0.66–0.99); lower average ONH-RNFL (OR = 0.75; 95% CI: 0.59–0.97); lower inferior ONH-RNFL (OR = 0.78; 95% CI: 0.62–0.98); and lower temporal ONH-RNFL (OR = 0.81; 95% CI: 0.66–0.99). When comparing MOPP and SOPP, a higher SOPP is less likely to result in lower GCC and ONH-RNFL thickness.

## 4. Discussion

Our study sought to investigate the effect of OPP on retinal thickness in a sample of young people with presumed systemic hypotension (PSH). The comparison of retinal thickness measurements between hypotensive and normotensive participants showed there were no significant differences in ONH- RNFL thickness and GCC thickness at the macula. However, the study established valuable associations between MOPP and SOPP within these retinal areas.

In comparison with participants with lower MOPP and SOPP, participants with higher MOPP and SOPP are associated with decreased odds of having lower total GCC thickness. This implies that higher MOPP and SOPP increase the likelihood of increased total GCC thickness. Furthermore, higher SOPP is associated with decreased odds of having lower superior hemisphere GCC thickness, as compared to participants with lower SOPP, thus suggesting protection from higher MOPP and SOPP in maintaining GCC total and superior hemispheric thickness at the macula.

The significant associations between ONH-RNFL thickness and SOPP showed that higher SOPP is associated with the likelihood of increased average ONH-RNFL thickness, and inferior and temporal ONH-RNFL thickness, when compared with participants with lower SOPP. These associations indicate the importance of maintaining increased perfusion pressure to avoid possible retinal thinning at the retinal areas.

The relationship of OPP to retinal thickness indicated that MOPP has a greater effect on GCC compared to the effect of MOPP on ONH-RNFL thickness. It was also found that higher SOPP will result in higher GCC and ONH-RNFL thickness in those with PSH when compared to MOPP. This association with GCC may be explained by RGC extra retinal associations [7].

In agreement with the Los Angeles Latino Eye Study [11] in which SOPP ≤ 80 mmHg was associated with a higher prevalence of primary open-angle glaucoma (POAG), our sample of presumed systemic hypotensives showed SOPP to be 75.77 mmHg. Similarly, the Barbados Eye Study [12] found that the incidence of POAG was highest with SOPP < 101.3 mmHg with an increased risk of 2.6 times for POAG. The above-mentioned comparison shows that our sample of presumed systemic hypotensives may also be at risk of POAG. MOPP (62.54 mmHg) was found to be statistically significant for our sample of systemic hypotensives with an increased risk for total GCC thinning. However, our MOPP was not similar to values found in the Latino and Barbados Eye Studies.

The present study showed a statistically significant association between MOPP and SOPP with GCC and ONH-RNFL thickness as shown in the logistic regression analysis. Although the Los Angeles Latino Eye Study [11] and Barbados Eye Study [12] did not measure retinal thickness, the similarities between our study and these studies make them comparable in the reduction of SOPP and may serve as a basis for the possible retinal thinning related to SOPP. In our study, since SOPP was significantly decreased in PSHs, and significant associations were found with GCC and ONH-RNFL thinning, this may indicate that these participants could be at risk of retinal thinning with reduced OPP, which could be related to glaucomatous structural changes.

MOPP was found to be statistically significant in this study. However, it was not comparable to the Los Angeles Latino Eye Study [11] or the Barbados Eye Study [12]. This could explain why no associations were seen between MOPP and DOPP and GCC and ONH-RNFL thickness. Since there is a lack of studies showing the effects of GCC and ONH-RNFL thinning as a result of reduced ocular perfusion pressure, no comparison can be made concerning the statically significant reduction in MOPP related to total GCC thickness. As this observation is occurring at the macula, it may suggest an early onset change that precedes ONH changes, which may be directly related to functional changes in vision.

Our study findings may have value as useful predictors and as an early sign for retinal morphology in a young sample of PSH who did not show any significant retinal thickness disruption but displayed reduced OPP and a decreased risk of RNFL and GCC thinning by increasing ocular perfusion pressure. In light of the above studies, PSH patients presenting with low BP and low OPP could, in the future, develop glaucoma; although in this study, they may be protected by age.

Constitutional hypotension refers to chronically reduced BP, independent of any pathological factors. People with low BP may present as an at-risk group and may go clinically undiagnosed until patients complain of symptoms. Cardiologists do not recognise systemic hypotension as a risk, and there is a lack of clinical guides for the diagnosis of low BP [36]. However, chronic systemic hypotension may cause damage to end organs, including the eye [37]. Constitutional hypotension is known to cause symptoms, such as dizziness and fatigue, and has been found to affect quality of life [4]. Wessely et al. [19] studied the symptoms of feeling faint and dizziness in participants with low BP. Particularly females and those at the extremes of the spectrum (the youngest and oldest participants), were most vulnerable. Females have also been found to be significantly associated with low BP and tiredness [22]. In middle-aged men, symptoms of dizziness, fatigue, and impaired mental well-being were found [20,21]. Tracking of these symptoms may be used to monitor those with chronically reduced blood pressure.

The ganglion cell complex (GCC) comprises the RNFL, the ganglion cell layer, and the inner plexiform layer. Since the GCC is made up of axons, cell bodies, and dendrites of the ganglion cell layer, it assists in early optic neuropathy detection [38]. Kergoat et al. [39] found that the innermost layers of the retina are most sensitive to low-level systemic hypoxia. Progressive RGC death leads to optic nerve head (ONH) degeneration and ultimately, the vision loss evident in glaucoma sufferers [40]. This indicates that assessing the GCC and RNFL may be beneficial in those with PSH as changes may be detected at an earlier stage.

Hashim et al. [41] studied the prevalence of glaucoma in OSAS patients. RNFL measurements showed that 87.5% of participants with severe OSAS had glaucoma. The study concluded that the alteration in OBF in OSAS might have resulted in glaucoma. Since OSAS affects retinal blood flow and perfusion pressure, which is similarly found in those with hypotension, a comparison may be made between our sample of PSH and those with sleep apnoea. Zheng et al. [10] studied the classification of abnormalities of glaucoma in those with and without glaucoma. It was found that the superior-temporal and inferior-temporal RNFL thickness yield the best diagnostic performance for glaucoma detection. Kargi et al. [18] found the thinning of the ONH-RNFL to be a result of loss of ganglion cells secondary to hypoxia, as well as hypoxemia, induced by OSAS by measuring oxygen saturation. The study found a significant difference in the mild and severe OSAS group, compared to controls, in the average superior and inferior RNFL. In another OSAS study by Zengin et al. [42], a reduction in RNFL thickness in the superior, inferior, and nasal RNFL thickness was found between the first and last measurements taken. This may indicate changes in RNFL thickness as a result of reduced OBF in OSAS. Wang et al. [43] studied RNFL thickness in those with OSAS, and a significant difference was found between controls and OSAS in the RNFL superior and inferior quadrants.

In the present study, although no significant thinning was found in the GCC and RNFL, the study did show that the thinning of the total GCC and superior hemisphere and average, inferior, and temporal RNFL layers are likely in our sample of PSH participants. This may agree with thinning found in the abovementioned studies, especially in the inferior RNFL. Our study was conducted on a young population, whereas the above studies were conducted on an older population, which may be more susceptible to changes. Our study had younger participants in comparison to Kargi et al. [18], which had a study sample of with an average age of 45.1 years compared to our 23.53 years. The Los Angeles Eye Study [11] and the Barbados Eye Study [12] had similar mean ages, 65 years, which also reinforces the fact that, in a younger age group, lowered OPP may be a harbinger of retinal loss. Considering the increased risk of glaucoma with age, it could also be possible that people with PSH may have an increased susceptibility to POAG. Furthermore, the younger age profile of our sample may have precluded the finding of any significant RNFL loss.

The focal retinal loci indices that are susceptible to reduction in MOPP and SOPP in PSH are certain noteworthy tracking sites for progressive changes with time in PSH. The risk of optic neuropathy may be a concern at an older age, and the systemic control of hypotension may also require clinical intervention.

The limited sample size precludes widespread generalisation. The sample size was projected based on studies involving obstructive sleep apnoea syndrome and prevalence estimates from studies on low systemic hypotension [18,44]. This was a because of a lack of previous studies on systemic hypotension and the retina. Other limitations of the study included only accessing a younger age group with a female-gender-biased sample, which may also highlight this gender’s vulnerability to the condition. This may be in agreement with other studies that found females to predominate those with chronic hypotension [22,45]. Akahoshi et al. [46] studied the characteristics of chronic hypotension and found low SBP, low haemoglobin, low BMI, and low muscle mass to be significantly associated with hypotension. Thus, recording BMI, as well as anaemia, may be useful in profiling. However, no participants were recorded as being on iron tablets for anaemia. The inclusion of older participants, with and without POAG, as well as males, would build on the findings of our study.

Since this study included South African participants from different ethnic backgrounds, it is important to note that the prevalence of glaucoma may differ. Bonnemaijer et al. [47] conducted a multi-ethnic comparative study in Sub-Saharan Africans, and it was noted that a more progressive course of POAG is noted in this group compared to Europeans. The Barbados Eye Study [12] included black participants and found that the relative risk for open-angle glaucoma increased threefold with lower perfusion pressure. This may indicate that the participants of the present study may be more likely to present with more progressive signs of glaucoma, further reinforcing the likelihood of glaucomatous changes. One of the limitations of the present study is that the ethnic background was not taken into consideration. However, a follow-up study may provide an insight into the increased risk of glaucoma, as well as the progression of GCC and RNFL thinning in these participants of different ethnic backgrounds.

To obtain accurate BP and IOP readings, the setup and method of measurement were maintained. To factor in diurnal variations, BP and IOP readings were obtained between 9:00 and 10:00 and 15:00 and 16:00. BP and IOP have been found to peak in the morning hours and trough in the afternoon hours [25,26,27].

The Omron M7 Intelli is a clinically validated automatic blood pressure monitor and is approved by the European Society of Hypertension (ESH) International Protocol [45]. A study comparing automated BP measurements to ambulatory BP in patients with hypertension have shown a close agreement between the two types of BP measurements [48]. However, we recommend a larger scale study with twenty-four-hour ambulatory BP monitoring, thus recording circadian changes including nocturnal dips in BP. By doing so, the likelihood of lower nocturnal OPP measurements due to a nocturnal dip in BP may amplify the diurnal observations in our study. The Icare tonometer is comparable to the gold standard Goldmann tonometer, which also accounts for the extremes of IOP measurements [33,34]. Repeating the study with Goldmann applanation tonometry may improve the strength of our findings. This may take into account the effects of central corneal thickness, with rebound tonometry, compared to applanation tonometry. The Optovue iVue OCT used in the study prevented the interrogation of all layers of the retina, which may have precluded assessing other possibly vulnerable layers and zones. It also lacked the angio-OCT facility, thus preventing the closer observation of retinal vascular circulation for a more direct assessment of perfusion. Ophthamodynamometry has been used for a direct measurement of OPP. However, it is a difficult and cumbersome technique with the potential for error. Most ophthalmodynamometers have thus been abandoned in clinical practice. More modern methods of measurement include colour Doppler imaging as a technology of choice because of its low invasiveness and high reliability [49].

Furthermore, a follow-up study, including those with and without POAG, to track changes of ONH-RNFL thickness over time may be significant and allow changes to be detected at an early stage. This study compared the subfields of the ONH-RNFL between the two groups. However, this can be further evaluated by assessing the superior nasal, superior temporal, upper nasal, and upper temporal fields and similarly, the inferior and lower fields. The inclusion of angio-OCT may allow for a functional assessment of retinal perfusion and will complement the structural assessment of our study. The inclusion of colour Doppler imaging will assist in identifying eyes that are more likely to suffer further visual field deterioration [49].

## 5. Conclusions

The study found that a reduced OPP as a result of PSH has proven to be significant, although no significant changes in retinal thickness were found in comparison to the control group. The study established that, by increasing perfusion, in particular MOPP and SOPP, there is a decreased risk of thinning in the total GCC thickness; superior GCC thickness; and the average, inferior, and temporal ONH-RNFL thickness. It was also established that increasing MOPP will have a greater effect on the prevention of thinning of the GCC when compared to ONH-RNFL. Increasing SOPP will have a greater effect on reducing thinning in the GCC and ONH-RNFL when compared to MOPP. We recommend close observation of OPP in those with PSH in general and, in particular, those with a genetic predisposition for primary open-angle glaucoma, as it is surmised that PSH may compound this susceptibility, in light of the challenges in the diagnosis of chronic systemic hypotension, which may go unnoticed.

## Figures and Tables

**Table 1:** The mean IOP, BP, and OPP measurements for both PSH (experimental) and normotensive (control) groups.

Group	Mean IOP ±SD (mmHg)	Mean Systolic BP ±SD (mmHg)	Mean Diastolic BP ±SD (mmHg)	Mean MOPP ±SD (mmHg)	Mean SOPP ±SD (mmHg)	Mean DOPP ±SD (mmHg)
Experiment *n* = 15 (30 eyes)	13.48 ± 2.27	90.99 ± 5.06	68.15 ± 4.80	62.54 ± 4.23	75.77 ± 9.10	55.93 ± 6.54
Control *n* = 14 (28 eyes)	14.83 ± 2.14	107.43 ± 5.73	72.57 ± 5.42	69.54 ± 5.80	91.52 ± 9.10	59.00 ± 8.47
Mean Difference (*p*-values)	*p* = 0.024 ^#^	*p* < 0.001 ^#^	*p* = 0.002 ^#^	*p* < 0.001 ^#^	*p* < 0.001 ^#^	*p* = 0.099 *

**^#^**: independent *t*-test, *: Mann–Whitney U-test.

**Table 2:** A comparison of retinal nerve layer thickness (microns) at the optic nerve head between the presumed systemic hypotensive (experimental) and normotensive (control) participants.

Group	OPTIC NERVE HEAD-RNFL THICKNESS (Microns)													
	RNFL Average		Superior RNFL		Inferior RNFL		Nasal RNFL		Temporal RNFL		Superior Hemisphere RNFL		Inferior Hemisphere RNFL	
	Mean ± SD	Mean Difference (Range)	Mean ± SD	Mean Difference (Range)	Mean ± SD	Mean Difference (Range)	Mean ± SD	Mean Difference (Range)	Mean ± SD	Mean Difference (Range)	Mean ± SD	Mean Difference (Range)	Mean ± SD	Mean Difference (Range)
Experimental *n* = 30 eyes	107.23 ± 7.47	1.07 (−3.55;5.69)	107.61 ± 10.62	−0.34 (−6.31;5.63)	103.68 ± 10.51	n/a	79.94 ± 8.16	n/a	75.03 ± 8.14	−0.12 (−4.68;4.44)	136.67 ± 13.08	6.52 (−2.17;15.21)	136.26 ± 13.29	0.26 (− 6.84;7.35)
Control *n* = 28 eyes	106.16 ± 9.87		107.95 ± 11.86		104.21 ± 8.83		82.160 ± 14.28		75.15 ± 9.04		130.15 ± 18.26		136.00 ± 12.31	
*p*-value		0.643 ^#^		0.910 ^#^		0.626 ^¥^		0.263 ^¥^		0.958 ^#^		0.138 ^#^		0.943 ^#^

Mean difference: difference of means between control and experimental; (−) implies reduction. SD: standard deviation. **^#^**: independent *t*-test, **^¥^**: Mann–Whitney U-test (mean difference not applicable; comparing the difference of medians).

**Table 3:** A comparison of the ganglion cell complex (GCC) (microns) at the macula between the experimental (PSH) and control (normotensive) participants.

Group	Macula-GCC Thickness (Microns)					
	GCC Total		GCC Superior Hemisphere		GCC Inferior Hemisphere	
	Mean ± SD	Mean Difference (Range)	Mean ± SD	Mean Difference (Range)	Mean ± SD	Mean Difference (Range)
Experimental *n* = 30 eyes	99.90 ± 6.57	0.40 (−3.26;4.05)	99.444 ± 7.00	0.15 (−3.86;4.15)	100.533 ± 6.292	0.61 (−2.78;4.00)
Control *n* = 28 eyes	99.59 ± 7.21		99.296 ± 8.088		99.926 ± 6.479	
Independent *t*-test		*p* = 0.83		*p* = 0.94		*p* = 0.72

Mean difference: difference of means between control and experimental; (−) implies reduction. SD: standard deviation.

**Table 4:** The significant associations between OPP and retinal thickness in young people with presumed systemic hypotension.

	Retinal Thickness														
	Macula GCC						ONH-RNFL								
	Total			Superior Hemisphere			Average			Inferior			Temporal		
	OR	CI	*p*-Value	OR	CI	*p*-Value	OR	CI	*p*-Value	OR	CI	*p*-Value	OR	CI	*p*-Value
**MOPP**	0.51	0.27; 0.97	0.039												
**SOPP**	0.79	0.64; 0.98	0.035	0.78	0.62; 0.99	0.049	0.11	0.01; 0.89	0.027	0.08	0.01; 0.73	0.017	0.26	0.04; 0.56	0.036

OR: odds ratio; CI: confidence interval; GCC: ganglion cell complex; ONH-RNFL: optic nerve head–RNFL; MOPP: mean ocular perfusion pressure; SOPP: systolic ocular perfusion pressure. No associations were observed for DOPP (diastolic ocular perfusion pressure).

## Data Availability

The data that support the findings of this study are available on request from the corresponding author.
